# Congenital aortic valve stenosis: from pathophysiology to molecular genetics and the need for novel therapeutics

**DOI:** 10.3389/fcvm.2023.1142707

**Published:** 2023-04-28

**Authors:** Jun Yasuhara, Karlee Schultz, Amee M. Bigelow, Vidu Garg

**Affiliations:** ^1^ Center for Cardiovascular Research, Abigail Wexner Research Institute, Nationwide Children’s Hospital, Columbus, OH, United States; ^2^ Heart Center, Nationwide Children’s Hospital, Columbus, OH, United States; ^3^ Medical Student Research Program, The Ohio State University College of Medicine, Columbus, OH, United States; ^4^ Department of Pediatrics, The Ohio State University, Columbus, OH, United States; ^5^ Department of Molecular Genetics, The Ohio State University, Columbus, OH, United States

**Keywords:** congenital aortic valve stenosis, bicuspid aortic valve, human genetics, cardiac development, animal models

## Abstract

Congenital aortic valve stenosis (AVS) is one of the most common valve anomalies and accounts for 3%–6% of cardiac malformations. As congenital AVS is often progressive, many patients, both children and adults, require transcatheter or surgical intervention throughout their lives. While the mechanisms of degenerative aortic valve disease in the adult population are partially described, the pathophysiology of adult AVS is different from congenital AVS in children as epigenetic and environmental risk factors play a significant role in manifestations of aortic valve disease in adults. Despite increased understanding of genetic basis of congenital aortic valve disease such as bicuspid aortic valve, the etiology and underlying mechanisms of congenital AVS in infants and children remain unknown. Herein, we review the pathophysiology of congenitally stenotic aortic valves and their natural history and disease course along with current management strategies. With the rapid expansion of knowledge of genetic origins of congenital heart defects, we also summarize the literature on the genetic contributors to congenital AVS. Further, this increased molecular understanding has led to the expansion of animal models with congenital aortic valve anomalies. Finally, we discuss the potential to develop novel therapeutics for congenital AVS that expand on integration of these molecular and genetic advances.

## Introduction

Congenital heart disease (CHD) is the most frequently occurring birth defect with an incidence of 0.8%–1.2% among live births (1, 2). Congenital aortic valve stenosis (AVS) has an incidence of 3.8–4.9 per 10,000 live births, representing ∼3%–6% of all CHD (1–3). Congenital AVS occurs more commonly in males as compared to females, with a reported ratio ranging from 3 to 5:1 (4, 5). It is defined as an obstruction of the aortic valve orifice due to a congenital valve malformation, which could be in the form of a bicuspid aortic valve (BAV), unicuspid aortic valve, or fused or malformed aortic valve cusps (6, 7) Associated CHD is found in approximately 20% of patients with congenital AVS, including ventricular septal defect (VSD), coarctation of the aorta (CoA), and patent ductus arteriosus (8). In this review, we aim to describe our understanding of aortic valve development with a particular focus on the embryology, anatomy and pathology relevant to congenital AVS. Further, we discuss the clinical characteristics of congenital AVS, including the natural history and current treatment options. Lastly, we highlight the molecular genetics of congenital AVS and discuss the prospects for the development of future potential therapeutics.

### Embryology

**Figure 1:**
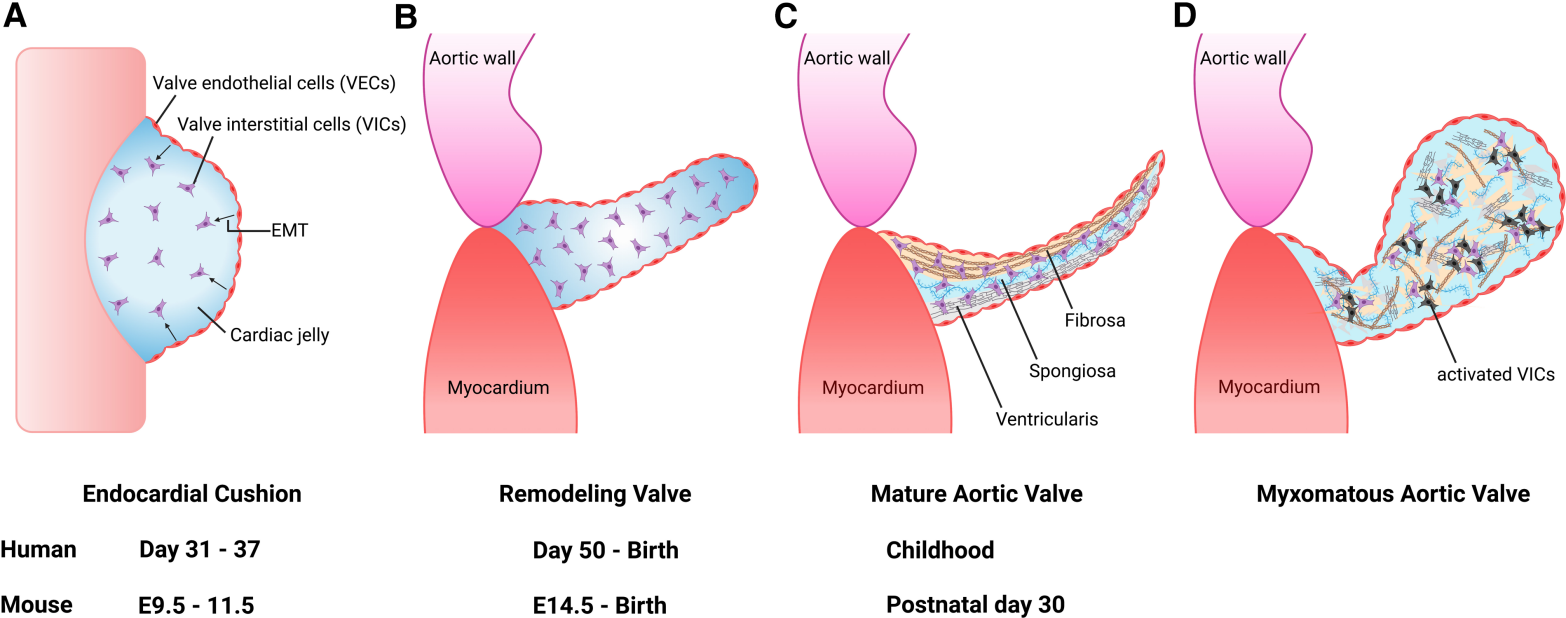
Aortic valve development and progression to myxomatous aortic valve. (**A**) Valve development begins with endocardial cushion formation. Valve endothelial cells (VECs) lining the cushion undergo endothelial-to-mesenchymal transformation (EMT) to differentiate into valve interstitial cells (VICs) between 7 and 9 weeks gestation in humans, and embryonic day (**E**) 9.5 and E11.5 in the mouse. (**B**) The outflow tract endocardial cushions undergo remodeling into mature valve leaflet. (**C**) Mature valve layers consist of fibrosa, spongiosa, and ventricularis. (**D**) Myxomatous alterations associated with leaflet thickening, activated VICs (black cells), disorganized extracellular matrix, diffuse accumulation of proteoglycans and fragmentation of collagen and elastin fibers.

Heart development occurs during the first trimester of pregnancy in humans from the embryonic gestational ages of 6 to 9 weeks. Here, we focus on semilunar valve development and refer the reader to comprehensive reviews on cardiac morphogenesis for details on this process (9, 10). Development of semilunar valves, which include the aortic valve and pulmonary valve, initiates between 7 and 9 weeks of gestation in the human embryo, and development of mature valve cusps continues after birth (11). This process has been well studied in mouse embryogenesis and is detailed here (12, 13). The primitive linear heart tube consists of an outer layer of myocardium and an inner layer of endocardium at embryonic day (E) 8.0. By E9.5, the heart has undergone rightward looping and is composed of the following 4 segments: the atrium, atrioventricular canal (AVC), ventricle, and outflow tract (OFT). During this time, a subset of endocardial cells forms swellings known as endocardial cushions within the AVC and embryonic cardiac OFT. In response to molecular signals from the adjacent myocardium, endocardial cells covering the cushions undergo endothelial-to-mesenchymal transformation (EMT) between E9.5 and E11.5 (Figure 1A). These mesenchymal cells migrate into the cardiac jelly and proliferate to occupy the endocardial cushions in the proximal OFT, while in the distal OFT the cardiac neural crest cells (CNC) are the major contributor. By E11.5, the AVC and OFT cushions have completed cellularization. After E11.5, the AVC and OFT cushions rapidly grow and remodel. This involves both apoptosis of valvular interstitial cells (VICs) as well as dynamic ECM arrangement (Figure 1B). Complex molecular networks tightly regulate each step of EMT, including the transforming growth factor-*β* (TGF-*β*) (14, 15), bone morphogenetic protein (BMP) (15, 16), WNT (17, 18), Notch (19, 20) and vascular endothelial growth factor A (VEGF) signaling pathways (21, 22). Although EMT is required for pooling mesenchyme valve precursors within the endocardial cushions, other cell lineages also play an essential role, including the CNC and secondary heart field (SHF) cells (23–27). CNC cells occupy the distal outflow tract cushions after migration from the aortic sac. By E12.5, the distal outflow tract is divided into the into the aorta and pulmonary artery by the aortopulmonary septum. On the other hand, the proximal outflow tract cushion contains mostly EMT-derived mesenchymal cells. During cushion development, the two cushions fuse at the distal-proximal boundary, where neural crest- and endothelium-derived mesenchymal cells meet, and the proximal outflow tract is separated into two ventricular outlets. The non-fused cushions then undergo extensive ECM remodeling and morphological sculpting and extend to become the coronary apex of the aortic valve. Mature left and right coronary cusps are derived primarily from endothelium-derived mesenchymal progenitor cells, with little contribution from CNC cells, while non-coronary cusp contains cells of the SHF lineage (28, 29).

### Aortic valve anatomy

The aortic valve is located between the left ventricle (LV) and ascending thoracic aorta (6). Like all valves in the heart, it serves to maintain unidirectional flow. The aortic valve is avascular and is connected to the aortic root by the fibrous annulus (30). Each leaflet is named according to the location corresponding to the coronary artery ostia and are accordingly referred to as the right coronary cusp, left coronary cusp and noncoronary cusp (31). Each cusp is composed of three layers of extracellular matrix (ECM) components that are oriented relative to blood flow, including the fibrosa comprised of collagen, the proteoglycan-rich spongiosa, and the elastin fiber-containing ventricularis (32) (Figure 1C). In addition, fibronectin and lamin are included in the aortic valve cusps as minor ECM components (33). The cellular composition of the aortic valve is comprised of VICs and valve endothelial cells (VECs). VICs are found within the interior of each cusp and important for ECM synthesis and homeostasis, while VECs form a protective cellular layer that surrounds each cusp. The communication between VICs and VECs *via* paracrine signaling is necessary to preserve ECM homeostasis and prevent disease (34–36).

### Aortic valve pathology

The pathologic features of diseased heart valves include myxomatous degeneration, which is characterized histologically by leaflet thickening, diffuse accumulation of proteoglycans and fragmentation of collagen and elastin fibers (Figure 1D) (37). Myxomatous valve disease is more commonly found in the mitral valve. It can be caused by congenital valve malformations, genetic abnormalities such as pathogenic variants in ECM genes, or can be acquired due to autoimmune diseases, progressive cardiovascular dysfunction/heart failure and infective endocarditis (38–40). Myxomatous valves also demonstrate an increased recruitment of pro-inflammatory macrophages and immunogenic ECM remodeling consistent with an inflammatory micro-environment (41). These findings suggest that macrophages play a role in the initiation and progression of myxomatous valve disease.

Aortic valves in infants with severe congenital AVS were reported to be described as “greatly thickened” and “nodular” and often had bicuspid morphology (42). Pathological and histological findings of congenital AVS demonstrated the primitive mesenchymal tissue, located between the valve endothelial layers, to be increased as well as containing a loose myxomatous ground substance. Further, the cells within the ground substance were described as spindle-shaped, similar to that seen in myxomatous valve tissue (Figure 1D). Interestingly, the valvular tissue resembled that of the endocardial cushions in the developing fetal heart. In normal heart development, loose connective tissue within the endocardial cushions is supposed to thin out and transition into dense, mature tissue. As a result, it is suggested this persistence of this loose embryonic connective tissue and likely its continued growth is a defining feature of congenital AVS.

Inflammation remains unclear as a contributor to congenital AVS. However, inflammation plays a significant role in many of the chronic diseases of adulthood, including cardiovascular disorders (43). Valvular inflammation can result in fibrosis, thickening, and calcification. Calcific aortic valve disease (CAVD) is the most common valvular condition in the developed world and increases in prevalence with age (44). Evidence of chronic inflammatory infiltrates in tissue exhibiting CAVD has been demonstrated along with a positive correlation between rate of progression and density of leukocytes (45). In the case of BAV, chronic inflammation may explain the earlier onset of disease, given that stenosis of a BAV is associated with increased inflammatory cells and vascularity in comparison to AVS in a tricuspid aortic valve (46). Together, these findings demonstrate that congenital AVS and adult-onset AVS may differ in regard to inflammation. There are multiple glycoproteins which regulate the aortic valve structure during development and are associated with progression (47, 48). N-glycosylation was found to be spatially regulated within the normal aortic valve and sialylated N-glycans were increased in pediatric end-stage congenital AVS (49). Collagen deregulation is a distinctive feature of congenital AVS, while the regulation of the collagen fibers in the aortic valve remains largely elusive. A recent study identified the collagen types and hydroxylated prolines (HYP) modifications, which are critical to stabilizing the triple helix of collagen, that are seen during human aortic valve development and at pediatric end-stage congenital AVS (50). Histological and proteomic analysis identified a unique region of high-density collagen present in pediatric end-stage congenital AVS and reported that specific collagen peptides were modified by HYP. In addition, network analysis identified BAMBI (BMP and Activin Membrane Bound Inhibitor) as a prospective regulator of the collagen interactome.

### Clinical characteristics, natural history and management of congenital AVS

Clinical presentations of congenital AVS vary widely, ranging from mild to critical, depending on the aortic valve morphology and the severity of AVS, but progress over time. During the fetal period, mild or moderate AVS leads to increased LV pressures and LV hypertrophy. Severe AVS results in severe LV hypertrophy and decreased flow through LV, which may ultimately lead to hypoplastic left heart syndrome (HLHS) (51–53). The fetus usually tolerates severe AVS, but symptoms can develop rapidly after birth. Critical AVS in neonates often presents with heart failure, cardiogenic shock, and other end organ dysfunction and can lead to death within the first weeks of life (54). Older children and adolescents with AVS tend to be asymptomatic with approximately 10% experiencing symptoms and signs of congestive heart failure, including dyspnea, angina, or syncope especially upon exercise (55). A combination of maximum aortic velocity (V_max_), mean pressure gradient (MPG), and aortic valve area (AVA) are used to assess the severity of stenosis as published in the most recent American College of Cardiology (ACC) and American Heart Association (AHA) guidelines (56).

The natural history of patients with congenital AVS shows that progressive obstruction is likely to occur by late adulthood. In childhood, significant progression was seen in one third of all medically managed patients in the Natural History Study (57). A follow-up study for 30 years found that the diagnosis of mild AVS before 6 months of age was associated with a significantly increased risk of requiring aortic valvotomy and balloon valvuloplasty with age (58). The likelihood that the stenosis remains mild was reported to be less than 20%, thus supporting the need for long-term follow-up of mild AS into adulthood. Recently, the probability of requiring balloon valvuloplasty is shown to be 20% in patients with catheter-measured peak pressure gradients less than 25 mmHg, and 40% and 70% in patients with gradients 25–49 mmHg and >50 mmHg, respectively (59). Notably, congenital AVS is a progressive disorder as the risk of morbid events such as heart failure, sudden death, and ventricular arrhythmia increase at a rate of 1%–1.5% per year, if left untreated (6, 59). Similarly, the risk of developing AVS in children with isolated BAV increases along with age (6). As with all CHD, bacterial endocarditis remains a potential complication of AVS, with an incidence of 27.1 per 10,000 person years (60). As congenital AVS is a progressive condition, guidelines have been developed which outline the indications for intervention. These interventions are limited to balloon valvuloplasty in the cardiac catheterization laboratory and transcatheter aortic valve replacement or surgical aortic valve replacement. We refer readers to recent clinical management guidelines from the ACC/AHA or European Society of Cardiology (ESC) for timing of intervention and different valve replacement options as these details are beyond the scope of this review (56, 61, 62).

### Molecular genetics of congenital aortic valve disease

Recent advances in genetic sequencing technologies, such as massively parallel sequencing, as well as interpretation of the clinical presentation and genetic variants, have made it easier for establishing a diagnosis and discovering new genetic etiologies for congenital aortic valve disease, including *ADAMTS19, SMAD6 and ROBO* gene family members (63, 64). Multiple human genetic abnormalities associated with syndromic and non-syndromic congenital aortic valve disease have been identified, including AVS and BAV (Tables 1, 2). However, compared to BAV, well established genetic contributors to congenital AVS are scarce. Common syndromes associated with congenital AVS are Turner syndrome and Jacobson syndrome. Turner syndrome is caused by chromosomal aneuploidies (Monosomy X) and associated CHD is observed in 30% of cases, including AVS, BAV, CoA and HLHS (65). Jacobsen syndrome is caused by terminal deletion of chromosome 11q and associated CHD is found in 56% of cases, including AVS, HLHS, CoA and VSD (66). In addition to Turner syndrome and Jacobsen syndrome, several common syndromes associated with BAV are known. Congenital heart valve anomalies associated with Trisomy 18 (Edwards syndrome) include BAV, bicuspid pulmonary valve and polyvalvular nodular dysplasia (67, 68). BAV is present in 1p36 deletion syndrome as well as Kabuki syndrome caused primarily by *KMT2D* and *KDM6A* variants (69–71). BAV and thoracic aortic aneurysm (TAA) have been also described in Marfan syndrome associated with *FBN1* variants (72) or Loeys-Dietz syndrome associated with *TGFBR1* and *TGFBR2* variants (73).

**Table 1:** Genetic syndromes associated with congenital aortic valve disease.

Syndrome	Gene	Location	Cardiac defects	Gene MIM	Reference
Turner syndrome	Unknown	45, X (monosomy X)	AVS, CoA, BAV, dilated Ao, HLHS	NA	(65)
Jacobsen syndrome	*ETS*	11q terminal deletion	AVS, HLHS, VSD, CoA, Shone's complex	164720	(66)
	*FLI1*			193067	
Edwards syndrome	Unknown	Trisomy 18	BAV, Bicuspid pulmonary valve, Polyvalvular nodular dysplasia	NA	(67, 68)
Kabuki syndrome	*KMT2D*	12q13.12	BAV, CoA, VSD, TOF, HLHS, TGA	602113	(69–71)
	*KDM6A*	Xp11.3		300128	
Marfan syndrome	*FBN1*	15q21.1	BAV, TAA, aortic dissection, mitral valve prolapse	134797	(72)
Loeys-Dietz syndrome	*TGFBR1*	9q22.33	BAV, TAA, aortic dissection, mitral valve prolapse	190181	(73)
	*TGFBR2*	3p24.1		190182	

AVS, aortic valve stenosis; BAV, bicuspid aortic valve; CoA, coarctation of the aorta; dilated Ao, dilated ascending aorta; HLHS, hypoplastic left heart syndrome; NA, not available; TAA, thoracic aortic aneurysm; TGA, transposition of great arteries; TOF, tetralogy of Fallot; VSD, ventricular septal defect.

**Table 2:** Human genes associated with congenital aortic valve disease.

Gene	Location	Cardiac defects	Gene MIM	Reference
*NOTCH1*	9q34.3	AVS, BAV, CAVD, HLHS, TOF, PS, VSD, CoA, TAA	190198	(74–82)
*SMAD6*	15q22.31	AVS, BAV, CoA, TAA	602931	(83)
*ROBO4*	11q24.2	AVS, BAV, ASD, TAA	607528	(84)
*VEGFA*	6p21.1	AVS, BAV, CoA, VSD, PDA, dilated Ao	192240	(85, 86)
*ADAMTS19*	5q23.3	AVS, Aortic valve insufficiency, BAV, subaortic stenosis, PVS, pulmonary valve insufficiency, mitral/tricuspid valve insufficiency	607513	(87, 88)
*GATA5*	20q13.33	BAV, ASD, VSD, DORV, TOF	611496	(89–91)
*GATA4*	8p23.1	BAV, ASD, VSD, AVSD, PS, TOF	600576	(92, 93)
*GATA6*	18q11.2	PTA, TOF, BAV	601656	(94–96)
*NKX2.5*	5q35.1	BAV, ASD, atrioventricular conduction delay, TOF, HLHS	600584	(97)
*NOS3*	7q36.1	BAV	163729	(98, 99)
*TAB2*	6q25.1	BAV, Aortic stenosis, subaortic stenosis, ASD, TOF, VSD, myxomatous mitral/tricuspid valves	605101	(100, 101)
*MAT2A*	2q11.2	BAV, TAA	601468	(102)

ASD, atrial septal defect; AVS, aortic valve stenosis; AVSD, atrioventricular septal defect; BAV, bicuspid aortic valve; CoA, coarctation of the aorta; dilated Ao, dilated ascending aorta; DORV, double-outlet right ventricle; HLHS, hypoplastic left heart syndrome; PDA patent ductus arteriosus; PS, pulmonary stenosis; PTA, persistent truncus arteriosus; PVS, pulmonary valve stenosis; TAA, thoracic aortic aneurysm; TOF, tetralogy of Fallot; VSD, ventricular septal defect.

For non-syndromic congenital AVS, only a few genes have been implicated. *NOTCH1* pathogenic variants were identified in individuals with left ventricular outflow tract (LVOT) malformations, including congenital AVS, CoA and HLHS (74, 75). *SMAD6* variants were observed in patients with AVS and BAV (83). *ROBO4* variants were also identified in individuals with AVS and atrial septal defect (ASD) (84). Furthermore, variants in *vascular endothelial growth factor-A* (*VEGFA*) were found in a patient with congenital tricuspid AVS and LVOT obstruction (85, 86). Moreover, *ADAMTS19* variants were found to cause a spectrum of congenital heart valve diseases, including AVS, aortic valve insufficiency, subaortic stenosis, pulmonary valve stenosis, pulmonary valve insufficiency and atrioventricular valve insufficiency (87, 88). However, no other genes have been reported as monogenic causes of congenital AVS. Previous studies reported that common variants in genes linked to cardiac development such as *ERBB4*, *BMP4*, and *ISL1*, may bestow risk for LVOT defects, including congenital AVS (103–105).

Taking another approach, genome-wide DNA methylation analysis identified significant alterations in CpG methylation at 59 sites in 52 genes for congenital AVS (106). A significant epigenetic change in the *APOA5* and *PCSK9* genes, which are known to be important in lipid metabolism, was also observed associated with AVS. It remains to be determined if pathogenic variation in the other 50 genes will be implicated in congenital AVS.

As BAV is often found in the setting of congenital AVS, there is likely overlap in the genetic etiologies of BAV and congenital AVS. BAV is widely acknowledged to have genetic contributors with a reported heritability of 89% (107). Insights into the genetic contributors of BAV were first provided from studies of familial BAV, where *NOTCH1* variants were discovered to segregate in familial aortic valve disease through linkage analysis, including BAV, AVS and CAVD (74). Since then, pathogenic variants in *NOTCH1* have been found to cause not only left -sided CHD, including BAV, CoA and HLHS (75, 76–79), but also other types of CHD such as tetralogy of Fallot (TOF) and VSD (80–82). The GATA family of zinc-finger transcription factors, particularly GATA4, GATA5, and GATA6, play essential roles in cardiac development. Pathogenic variation in *GATA5* is well characterized in human BAV (89–91), but *GATA5* variants have also been associated with a spectrum of CHD, including TOF, VSD, ASD and double outlet right ventricle (DORV) (108–110). *GATA4* pathogenic variants were identified in BAV cases (92) and in addition, the burden of rare variants in *GATA4* were shown to be significantly enriched in early-onset BAV (93). Although *GATA6* variants have been mainly implicated in conotruncal heart defects (94, 95), *GATA6* loss-of-function variants were identified in a family with BAV (96). Furthermore, a deleterious variant in *NKX2.5* was identified in a family with BAV (97), while pathogenic variants in *NKX2.5* have been reported in ASD along with atrioventricular conduction abnormalities, VSD, TOF and HLHS (111–113). The contribution of other candidate genes, such as *NOS3, TAB2,* and *MAT2A* has been also suggested in BAV (98–102).

### Mouse models of congenital aortic valve disease

**Figure 2:**
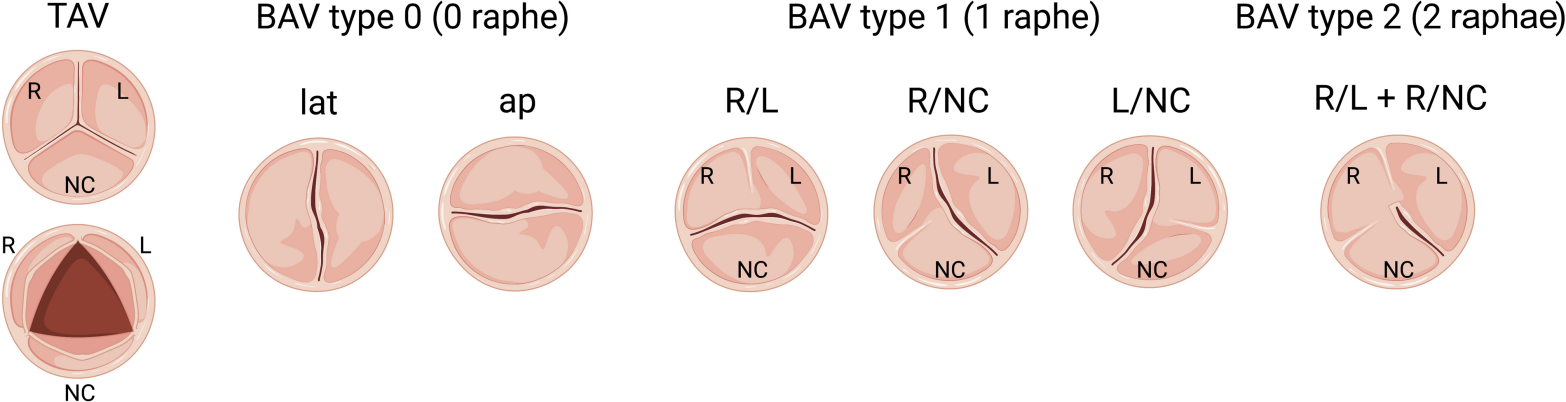
Schematic of morphologic phenotypes in bicuspid aortic valve (BAV). Schematic depiction of a normal tricuspid aortic valve (TAV) and three subtypes of BAV based on the raphe position relative to coronary artery origins: Type 0 (no raphe), Type 1 (one fibrous raphe) and Type 2 (two raphae). Type 1 is the most common, including BAV R/L (right-left fusion), R/NC (right-noncoronary fusion) and L/NC (left-noncoronary fusion), followed by Type 0, including lat (lateral arrangement of the free edge of the cusps) and ap (anterior-posterior arrangement of the free edge of the cusps), and the most infrequent is Type 2.

Since congenitally stenotic aortic valves have often been manipulated due to current management strategies, there is limited access to diseased human tissues to investigate mechanisms of disease initiation and progression. Accordingly, animal models serve an important role in understanding the genetic etiologies and progression of congenital AVS (114). A summary of reported genetic mouse models found to exhibit congenital aortic valve disease can be found in Table 3, including congenital AVS and BAV. Morphological phenotypes in BAV are summarized in Figure 2 (133). Although *NOTCH1* has been implicated in human aortic valve disease, *Notch1* haploinsufficiency causes CAVD and ascending aortic aneurysms in mice, but not congenital aortic valve abnormalities (134, 135). Interestingly, while mice which are homozygous for a null mutation in endothelial NOS (*Nos3*^−/−^) display a partially penetrant BAV at an incidence of ∼25% (117, 118), *Notch1*; *Nos3* compound mutant mice (*Notch1*^+/−^; *Nos3*^−/−^) display congenital aortic valve disease such as BAV and AVS at a penetrance of 64%. These aortic valve abnormalities are accompanied by additional cardiac outflow tract defects resulting in ≈65% lethality by postnatal day 10 (35, 115). Telomere shortening in *Notch1* haploinsufficient mice (*Notch1*^+/−^ *mTR*^G1–3^) elicit age-dependent tricuspid AVS and aortic valve calcification, however, early lethality is observed (116). In addition, cell-specific deletion or inactivation of mediators of Notch signaling pathway, such as *JAG1* or *RBPJ,* in mice demonstrate BAV at a penetrance of 47%–54% with high perinatal lethality (121, 122). Interestingly, no endocardial cushion defects are observed in these murine models of BAV targeting Notch ligands, suggesting that congenital aortic valve disease may result from a disruption in the process that occurs after EMT, potentially during the valve remodeling. Genetic deletion of *Gata5* in mice (*Gata5^−/−^*) lead to R/NC subtype BAV at a partially penetrance of 25% (119). *Gata6* haploinsufficient mice (*Gata6^+/−^*) develop R/L subtype BAV with incidence of 56% in males and 27% in females (120). Furthermore, SHF-specific deletion of *Gata6* within the *Isl1*-lineage recapitulated the BAV phenotype, suggesting the role of Gata6 in SHF during valve development. Heterozygous *Nkx2.5* knockout mice display a variety of cardiac phenotypes, depending on the genetic background, including BAV with AVS at a low penetrance of 8.2% (123). In addition to these mice, other genetic mouse models of congenital aortic valve disease have been described, whereas the majority have limitations with regards to a low penetrance or high lethality (84, 87, 124–132). Further studies are needed to generate murine models with a high penetrance that survive to adulthood, allowing us to validate the role of cardiac developmental genes in the etiology of aortic valve disease as well as investigate their role in disease progression and to serve as models to test novel therapies.

**Table 3:** Mouse models of congenital aortic valve disease.

Gene	Genotype of mouse model	Valve phenotype	Aortic valve disease penetrance	BAV subtype	Other cardiac defects	Lethality	Reference
*NOTCH1*	*Notch1*^+/−^; *Nos3*^−/−^	BAV, thickened AoV and PV	≈64%	NA	Ascending aortic dilation, VSD, overriding aorta	≈65%	(35, 115)
	*Notch1*^+/−^ *mTR*^G1–3^ (mTR^−/−^ generation 1–3)	AVS, Thickened AoV, CAVD, PVS	NA	None	Ascending aortic dilation, ASD, VSD	70%	(116)
*NOS3*	*Nos3^−/−^*	BAV	27–42%	R/NC	None	None	(117, 118)
*GATA5*	*Gata5^−/−^*	BAV	25%	R/NC	Mild LV hypertrophy	None	(119)
*GATA6*	*Gata6^+/−^*	BAV	56% males, 27% females	R/L	None	None	(120)
*ROBO4*	*Robo4^tm1Lex/tm1Lex^*	BAV, AVS, thickened AoV	18% males, 11% females	NA	Ascending aortic dilation	None	(84)
*ADAMTS19*	*Adamts19* ^KO/KO^	AVS, AR, thickened AoV, BAV	38%	NA	None	None	(87)
*JAG1*	*Nkx2.5Cre*+; *Jag1*^flox/flox^	BAV	47%	R/NC or R/L	VSD	94%	(121)
*RBPJ*	*Nkx2.5Cre*+; *Rbpj*^flox/flox^	BAV	54%	75% R/NC, 25% R/L	VSD, DORV	100%	(122)
*NKX2.5*	*Nkx2.5^+/−^*	BAV, AVS	8.2%	NA	ASD	None	(123)
*MATR3*	*Matr3^Gt−ex13^* heterozygotes	BAV	15%	NA	CoA, PDA, VSD, DORV	None	(124)
*EGFR*	*Egfr* ^Vel/+^	Unicuspid AoV, AVS, AR	38%	None	None	None	(125)
*BRG1*	*Nfatc1Cre*+; *Brg1*^flox/flox^	BAV, thickened AoV and PV	35%	67% L/NC, 33% R/NC	VSD	97%	(126)
*HOXA1*	*Hoxa1^−/−^*	BAV	24%	NA	VSD, TOF, IAA	NA	(127)
*ADAMTS5*	*Adamts5^−/−^*; *Smad2^+/−^*	BAV	41%	NA	Ascending aortic anomalies	None	(128)
*EXOC5*	*Nfatc1Cre*+; *Exoc5*^flox/+^	BAV, AVS, dysmorphic AoV	45%	80% R/NC, 20% L/N	VSD	None	(129)
*ALK2*	*Gata5Cre+; Alk2^FXKO^*	BAV	78%	NA	VSD	55%	(130)
*NPR2*	*Npr2^+/−^*	BAV, AVS, CAVD	9.4%	R/NC	Ascending aortic dilation, LV dysfunction	None	(131)
	*Npr2^+/−^*; *Ldlr*^−/−^						
*VGLL4*	*Vgll4* ^−/−^	Thickened AoV and PV	NA	NA	LV hypertrophy	89%	(132)
	*Tie2Cre*+; *Vgll4*^−/−^						

AoV, aortic valve; AR, aortic regurgitation; ASD, atrial septal defect; AVS, aortic valve stenosis; BAV, bicuspid aortic valve; CAVD, calcific aortic valve disease; CoA, coarctation of the aorta; DORV, double-outlet right ventricle; IAA, interrupted aortic arch; L/NC, left/noncoronary fusion; LV, left ventricular; NA, not available; NS, non-syndromic; PDA patent ductus arteriosus; PV, pulmonary valve; PVS, pulmonary valve stenosis; R/L, right/left fusion; R/NC, right/noncoronary fusion; SVAS, supravalvular aortic stenosis; TGA, transposition of great arteries; TOF, tetralogy of Fallot; VSD, ventricular septal defect.

### Clinical implications and future directions

Given the limited translation of molecular mechanisms found in animal models to human patients, effective pharmacologic therapies for congenital AVS remain elusive. The current standard treatment is transcatheter/surgical repair or replacement of the diseased valve. Recently, fetal aortic valvuloplasty has been performed in fetuses with AVS and evolving HLHS. However, outcomes for achieving biventricular circulation remain controversial and this has not been applied to isolated congenital AVS (136, 137). Thus, development of novel medical treatment for congenital AVS is essential, while pharmacological therapies are limited. One limited treatment option is statin therapy to treat elevated cholesterol levels. Statins have been demonstrated to reduce cardiovascular risk and prevent cardiovascular disease such as coronary artery disease (138, 139). Although some attempts have been made to utilize pharmacologic treatments such as statins to treat CAVD with associated AVS in adults, these studies did not show consistent beneficial effects for progression of AVS (140, 141). The pathophysiology of degenerative AVS in adults is similar to coronary artery disease, whereas atherosclerosis pathway may not play a significant role in the development of congenital aortic valve disease, including BAV. Therefore, statins have not been tested in children with aortic valve disease and the clinical impact on congenital AVS in children remains unknown. Increased understanding the molecular pathways regulating aortic valve development along with advances in genetic sequencing technologies have allowed for the discovery of new candidate genes for congenital aortic valve disease. In addition, genetic murine models of aortic valve disease have been generated and uncovered molecular pathways as potential therapeutic targets. Specifically, TGF-*β* signaling is activated in degenerative valves with ECM abnormalities and may be a potential therapeutic target, as TGF-*β* antagonists such as the angiotensin II type 1 receptor blocker, losartan, could prevent abnormal aortic root growth in a mouse model of Marfan syndrome (142). In addition, targeting monocyte-derived macrophages has emerged as a potential therapeutic approach to prevent myxomatous valve disease in Marfan syndrome mice (41). Recent studies by using single-cell RNA-sequencing, human induced pluripotent stem cell (iPSC) technology and machine learning, identified new pathogenic pathways and a new therapeutic candidate to prevent aortic valve disease in mouse models of CAVD, although the implications for congenital AVS are unknown (36, 143). Further, the molecular and genetic links between congenital aortic valve disease and adult CAVD need to be defined. Improved identification of human aortic valve disease genes, generation of murine models for clinically relevant congenital AVS, and new technologies such as single cell genomics, iPSC modeling and machine learning may reveal novel therapeutic targets to develop effective treatments for early intervention.

## Conclusions

Congenital AVS is a complex and progressive disease that affects children and adults throughout their lives. Although advances in transcatheter aortic valvuloplasty and transcatheter or surgical aortic valve implantation have improved morbidity and mortality in this patient population, pharmacologic therapies for congenital AVS remain elusive. Several potential therapeutic targets have been proposed in animal models to prevent the myxomatous valve disease by using next-generation sequencing, single-cell genomics, machine learning, cardiac organoid and bioengineering technologies. A continued escalation of our understanding in molecular genetics of congenital AVS has clinical implications as it will facilitate the development of new treatment options to prevent the progression or treat congenital AVS.
